# Mid-term outcome of transarterial embolization of renal artery pseudoaneurysm and arteriovenous fistula after partial nephrectomy screened by early postoperative contrast-enhanced CT

**DOI:** 10.1186/s42155-020-00160-z

**Published:** 2020-09-16

**Authors:** Satoru Morita, Yuka Matsuzaki, Takahiro Yamamoto, Kumi Kamoshida, Hiroshi Yamazaki, Tsuyoshi Tajima, Tsunenori Kondo, Toshio Takagi, Kazuhiko Yoshida, Kazunari Tanabe, Shuji Sakai

**Affiliations:** 1 Department of Diagnostic Imaging and Nuclear Medicine, Tokyo Women’s Medical University, 8-1, Kawada-cho, Shinjuku-ku, Tokyo, 162-8666 Japan; 2 School of Medicine, Tokyo Women’s Medical University, 8-1, Kawada-cho, Shinjuku-ku, Tokyo, 162-8666 Japan; 3 Department of Radiology, National Center for Global Health and Medicine, 1-21-1, Toyama, Shinjuku-ku, Tokyo, 162-8655 Japan; 4 Department of Urology, Tokyo Women’s Medical University Medical Center East, 2-1-10 Nishiogu, Arakawa-ku, Tokyo, 116-8567 Japan; 5 Department of Urology, Tokyo Women’s Medical University, 8-1, Kawada-cho, Shinjuku-ku, Tokyo, 162-8666 Japan

**Keywords:** Embolization, Renal artery, Pseudoaneurysm, Partial nephrectomy, Computed tomography

## Abstract

**Purpose:**

To retrospectively evaluate the mid-term outcome of transarterial embolization (TAE) of renal artery pseudoaneurysm (RAP) including arteriovenous fistula (AVF) after partial nephrectomy screened by early postoperative contrast-enhanced CT (CE-CT).

**Materials and methods:**

Eighty-two patients (7.0%) who underwent TAE after partial nephrectomy were reviewed, from 1166 partial nephrectomies performed over 6 years. In 18 patients (22.0%), TAE was performed emergently on the median postoperative day (POD) seven. In the remaining patients, elective TAE was performed on the median POD six for RAP detected by early postoperative CE-CT or that emerged on follow-up CE-CT.

**Results:**

In one patient (1.2%), TAE was performed twice because one of two RAPs could not be embolized during the first TAE, being successfully embolized at the second TAE after readmission with hematuria. Otherwise, no bleeding recurrence or RAPs were observed during the median 1354 follow-up days. Thus, the primary and secondary success rates of TAE were 98.8% (81 of 82 patients) and 100% (82 of 82 patients), respectively. On angiography, the average number of lesions was 1.7 ± 0.9 and the average RAP size was 12.8 ± 6.0 mm. The shapes of the lesions varied: oval-round 60, oval-round + AVF 36, irregular + AVF 14, AVF 12, irregular 10, disruption 4, and extravasation 3. No major complications were observed. The median inpatient days after TAE were two. No estimated glomerular filtration rate deterioration was observed (64.6 ± 18.6 vs. 64.2 ± 18.4 mL/min/1.73 m^2^, *p* = 0.902).

**Conclusion:**

TAE is largely effective and safe for treating bleedings or RAPs, including AVFs, after partial nephrectomy, as screened by early postoperative CE-CT.

## Introduction

Partial nephrectomy is a standard surgical procedure for small renal tumors to preserve renal function after surgery (Campbell et al. 2009; Ljungberg et al. 2010). Laparoscopic or robot-assisted laparoscopic partial nephrectomy has increasingly been used because of its reduced invasiveness (Yang et al. 2014; Aboumarzouk et al. 2012; Patel et al. 2013). However, studies have reported a higher incidence of hemorrhage after laparoscopic surgery, 1.2% to 7.5% (Yang et al. 2014; Netsch et al. 2010; Gill et al. 2007; Montag et al. 2011; Nadu et al. 2009; Takagi et al. 2014; Omae et al. 2015), when compared with that in open partial nephrectomy, 0.4% to 1.6% (Netsch et al. 2010; Gill et al. 2007; Albani and Novick 2003; Ghoneim et al. 2011). Renal artery pseudoaneurysm (RAP) formation causes hemorrhage and its presentation is often delayed to approximately 2 weeks after surgery (Montag et al. 2011; Albani and Novick 2003; Ghoneim et al. 2011; Hyams et al. 2011; Cohenpour et al. 2007). It has been shown that the incidence of RAP, 15%–20%, is higher than that previously reported, when screening by contrast-enhanced computed tomography (CE-CT) in the early postoperative period (Takagi et al. 2014; Omae et al. 2015). In addition, we show that transarterial embolization (TAE) of RAP detected on early postoperative screening by CE-CT can prevent delayed hemorrhage, which occurred in 4.6%–4.7% of cases without CE-CT screening and in 0.6% with CE-CT screening and TAE (Morita et al. 2015).

However, it is unknown whether delayed hemorrhage or recurrence of RAP, including arteriovenous fistula (AVF), can occur due to TAE or whether any adverse effects on the renal function can appear over a longer period after TAE. The purpose of this study was to retrospectively evaluate the efficacy and safety of TAE for RAP after partial nephrectomy in a large population of patients with mid-time follow-up who underwent early postoperative screening by CE-CT.

## Materials and methods

### Patients

This retrospective, single-institution study was approved by the institutional review board of our facility. Written informed consent for the individual patient data used in our analysis was waived because of the retrospective nature of the investigation. Between January 2012 and January 2018, 1168 partial nephrectomies were performed for renal tumors in 1142 patients. Two patients were excluded because of their young age (< 18 years). Open, laparoscopic, or robotic partial nephrectomy was performed using the standard techniques previously described (Takagi et al. 2014; Omae et al. 2015; Gill et al. 2002; Rogers et al. 2008). After 1166 partial nephrectomies, 82 patients (7.0%) underwent TAE. The clinical characteristics of these patients are summarized in Table 1.

**Table 1:** Patient characteristics

Age, years (median, range)	61 (26–81)
Sex (Male:Female)	63:19
Partial nephrectomy	Robotic 45 (54.9%), laparoscopic 16 (19.5%), open 21 (25.6%)
Maximum diameter of the tumor (mm)	30.4 ± 16.0
Pathological diagnosis^a^	Clear cell RCC 64 (78.0%), chromophobe RCC 2 (2.4%), papillary RCC 2 (2.4%), angiomyolipoma 7 (8.5%), oncocytoma 4 (4.9%), and others 3 (3.7%)
In RCC (*n* = 68)	
Pathological tumor stage^b^	pT1a 52 (76.5%), pT1b 14 (20.6%), pT2a 2 (2.9%)
Tumor complexity^c^	Low 29 (42.6%), intermediate 29 (42.6%), high 10 (14.7%)
Symptoms before TAE	Progressive anemia 24, severe hematuria 17, decrease in blood pressure 5, bleeding from drainage tube 5, severe pain 3

*RCC* Renal cell carcinoma, *TAE* Transarterial embolization. Classified according to the 2016 World Health Organization classifications^a^, 2009 TNM classification^b^, and R. E. N. A. L nephrometry score^c^

### CT examinations

Scheduled early postoperative CE-CT was performed 2–5 days after surgery to screen for surgical complications, mainly RAP, in each patient, except for those having renal insufficiency, adverse reactions to contrast materials, or with severe asthma. Postoperative CE-CT was performed using 64-row detector scanners (Aquilion 64; Canon Medical Systems, Otawara, Japan) or 320-row detector scanners (Aquilion One or Aquilion One GENESIS Edition; Canon Medical Systems). The images were obtained at the following settings: pitch, 0.81–0.83; collimation, 0.5 mm; reconstruction thickness/interval, 1.0 mm/1.0 mm; and 120 kVp with automatic exposure control. The early arterial phase images were obtained by using a bolus-tracking contrast monitoring system after injecting the contrast material for 30 s. The amount of contrast material, 600 mgI/kg with a maximum of 150 ml and an injection rate of 5.0 ml/s, depended on body weight and iodine concentration (300, 350, or 370 mgI/ml was used).

### Indications for TAE

The CT images were evaluated by one of two interventional radiologists: S.M and T.T, each with more than 10 years of experience in TAE. Trans-axial images with 1.0 mm thickness and multiplanar reformation and maximum intensity projection images at any angle of the kidney were evaluated using a viewer (ShadeQuest/ViewR; Yokogawa Medical Solutions, Tokyo, Japan) with a three-dimensional workstation (Aquarius iNtuition, TeraRecon, Foster City, CA). In emergent cases with symptoms and RAP detected by emergent CE-CT, TAE was performed within 1 day. For RAP detected on CE-CT, the treatment strategy was decided according to the protocol described as follows in this paragraph (Morita et al. 2015). In general, TAE was performed within 3 days when an apparent RAP, round and > 5 mm in diameter, was detected. In some cases, minute, irregular, linear, or faint contrast enhancements, which were difficult to diagnose as RAP, were observed. In these cases, the radiologists judged whether embolization was required. In difficult cases, early follow-up CE-CT was performed 2 to 5 days during hospitalization or 1 to 2 weeks after discharge. If the findings became larger or apparent as RAP, TAE was performed subsequently.

### Allocation of patients for TAE

**Fig. 1:**
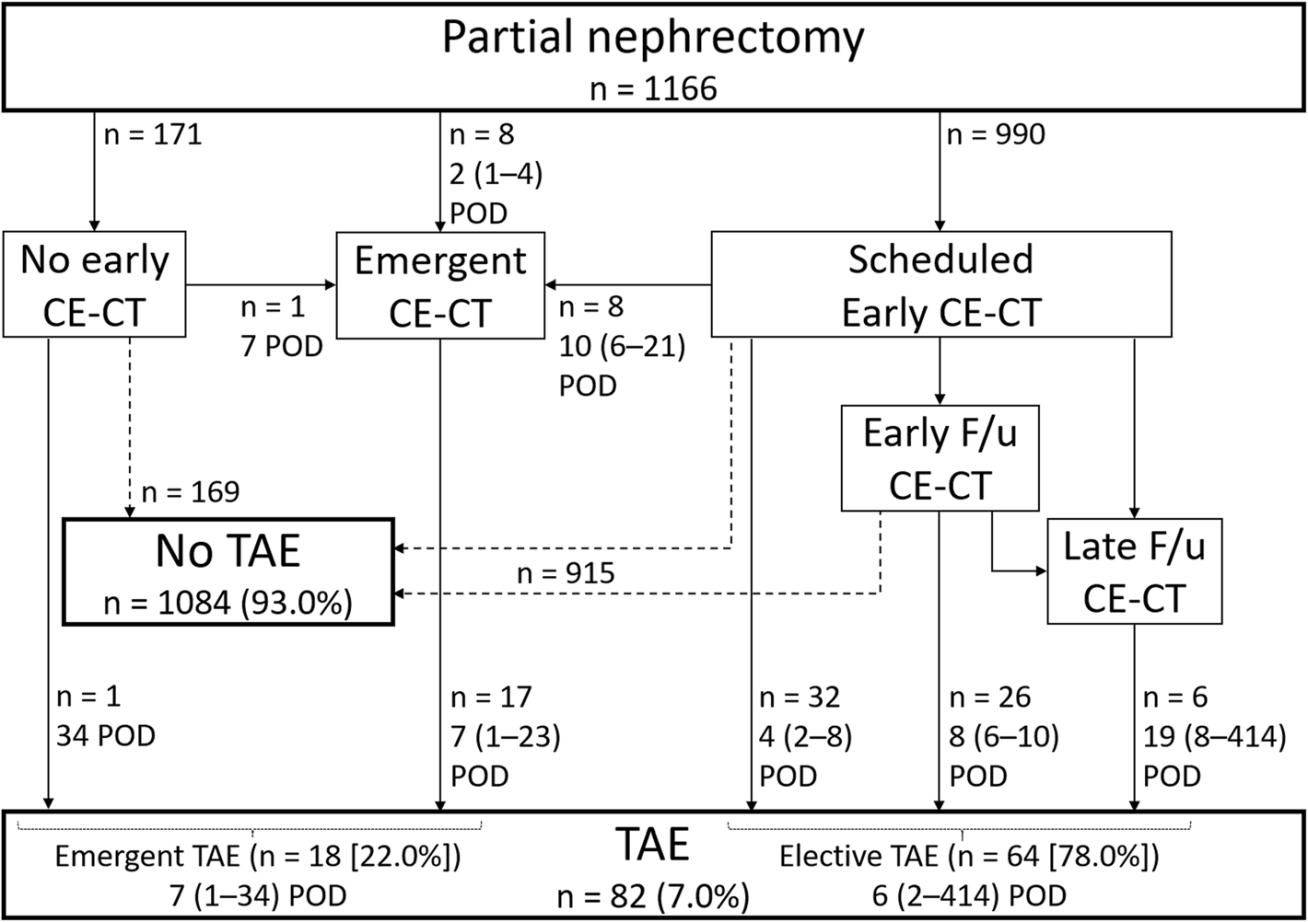
Flowchart of the allocation of patients for transarterial embolization (TAE). CE-CT = contrast-enhanced CT, POD = postoperative day; median (range), RAP = renal artery pseudoaneurysm, F/u = follow-up

A flowchart of the method of allocating patients for TAE is shown in Fig. 1. Overall, the median TAE postoperative day (POD) from surgery was six (range, 1–414). The median follow-up day after TAE was 1354 (range, 9–3220). TAE was performed emergently in 18 patients (22.0%) with symptoms as follows: severe hematuria 12, progressive anemia 11, decrease in blood pressure 5, severe pain 3, and bleeding from the drainage tube 1. One patient with renal insufficiency who had a sudden decrease in blood pressure and peritoneal hemorrhage on POD seven underwent TAE after confirming RAP by emergent CE-CT. One patient with renal insufficiency and sudden hematuria after discharge underwent TAE without CE-CT on POD 34. Eight patients underwent TAE after emergent CE-CT. The remaining eight patients underwent TAE after scheduled early CE-CT. In the other 64 patients (78.0%), elective TAE was performed for RAP that was either detected on early CE-CT or emerged by follow-up CE-CT.

### Embolization techniques

A 4-F sheath was inserted via the right femoral artery. Renal angiography was performed using a 4-F shepherd’s hook catheter. The RAP-feeding arteries were selected using microcatheters such as the 1.9-F Carnelian Marvel (Tokai Medical Products, Aichi, Japan) with 2.6-F Masters HF (Asahi Intecc Co., Ltd., Nagoya, Japan), 2.0-F Excelsior 1018 (Boston Scientific, Marlborough, Massachusetts), 2.5-F Renegade (Boston Scientific), or 2.0-F Masters Parkway (Asahi Intecc Co., Ltd). Embolization of the affected arteries was performed using microcoils while preserving the normal renal parenchyma as much as possible. Detachable microcoils, such as the Target coil (Stryker Inc., Tokyo, Japan), Penumbra coil (Penumbra Inc., Alameda, CA, USA) or the Interlocking detachable coil (IDC; Boston Scientific), were mainly used. Pushable coils such as the Tornado coil (Cook Inc., Bloomington, IN, USA) were also used. Gelatin sponges (Serescue; Nippon Kayaku Co., Ltd. Tokyo, Japan) or 1:4 mixtures of n-butyl cyanoacrylate and ethiodized oil (Lipiodol; Guerbet Japan, Tokyo, Japan) were used in some cases. In three cases of high-flow AVF, a balloon catheter (Selecon MP Catheter; Terumo, Tokyo, Japan) was used to prevent coil migration into the venous side. The angiographic findings and embolization methods were reviewed in consensus by two interventional radiologists (S.M and H.Y, each with more than 10 years of experience in TAE).

### Clinical outcome

Medical records were reviewed to identify bleeding and complications after TAE using the Society of Interventional Radiology (SIR) classification system for complications by outcome (Sacks et al. 2003). Laboratory data were evaluated, including the estimated glomerular filtration rate (eGFR) recorded pre-surgery, pre- and post- (on the day after) TAE, and most recently.

### Statistical analysis

The statistical analyses were performed using JMP 15 software (SAS Institute Inc., Cary, North Carolina). A *P* value of < .05 was considered statistically significant. The changes in eGFR were compared using the Student’s t-test.

## Results

**Fig. 2:**
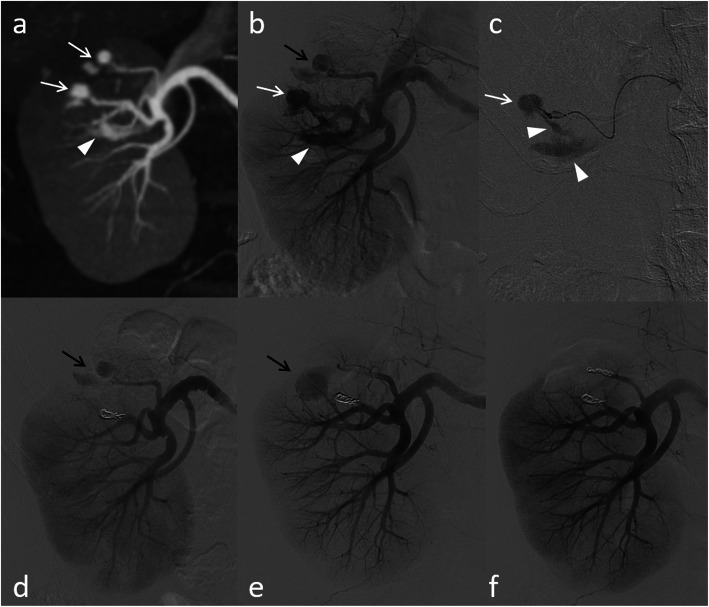
A case of undergoing transarterial embolization (TAE) twice in a 75-year-old man. **a** Two renal artery pseudoaneurysms (RAPs) (arrows) with arteriovenous fistula (AVF) (arrowhead) are observed on contrast-enhanced CT on postoperative day (POD) six. **b**, **c**, **d** Although one RAP (white arrows) with AVF (arrowheads) can be embolized using microcoils on POD eight, the other RAP (black arrows) cannot be embolized because of the difficulty of cannulation due to the acute angle of the branch. **e**, **f** The remaining RAP (black arrow), which is increased in size, is successfully embolized using microcoils at the second TAE after readmission with hematuria on POD 22

In one patient (1.2%), TAE was performed twice because one of the two RAPs could not be embolized during the first TAE on POD eight but was successfully embolized at the second TAE after readmission with hematuria on POD 22 (Fig. 2). Otherwise, no recurrence of bleeding or RAPs were observed. Thus, the primary and secondary success rates of TAE were 98.8% (81 of 82 patients) and 100% (82 of 82 patients), respectively.

**Fig. 3:**
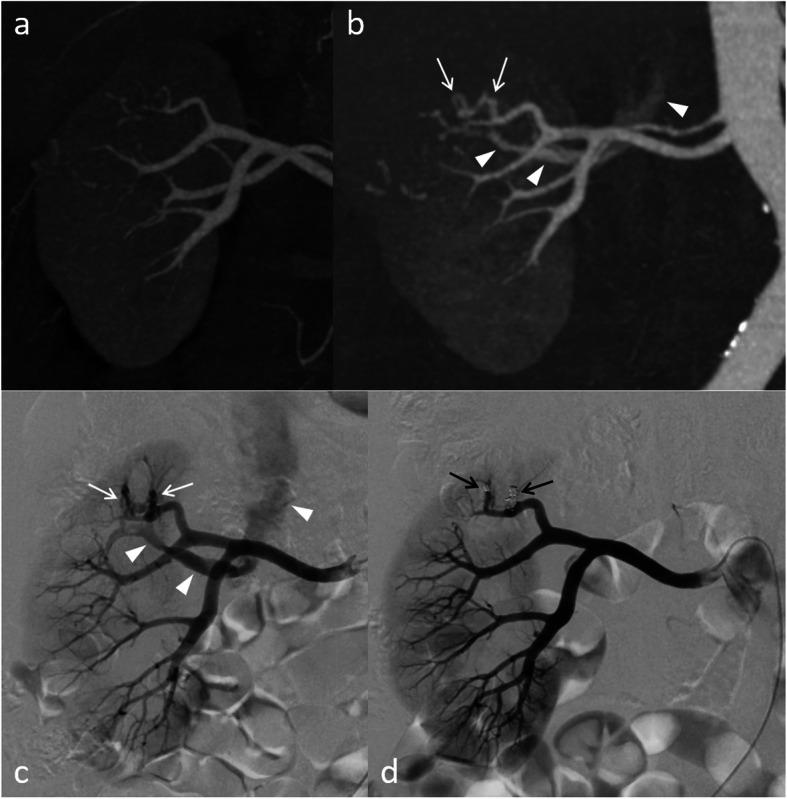
A case of two newly appearing arteriovenous fistulas (AVFs), with symptoms, in a 67-year-old woman. **a** No abnormal finding observed on early postoperative contrast-enhanced CT on postoperative day (POD) two. **b** Two AVFs (arrows) with early venous return (arrowheads) appear on CT obtained on POD 19, with hematuria after discharge. **c**, **d** Angiography showing the two AVFs (white arrows) successfully embolized using microcoils (black arrows), with early venous return (arrowheads) disappearing on POD 21

**Fig. 4:**
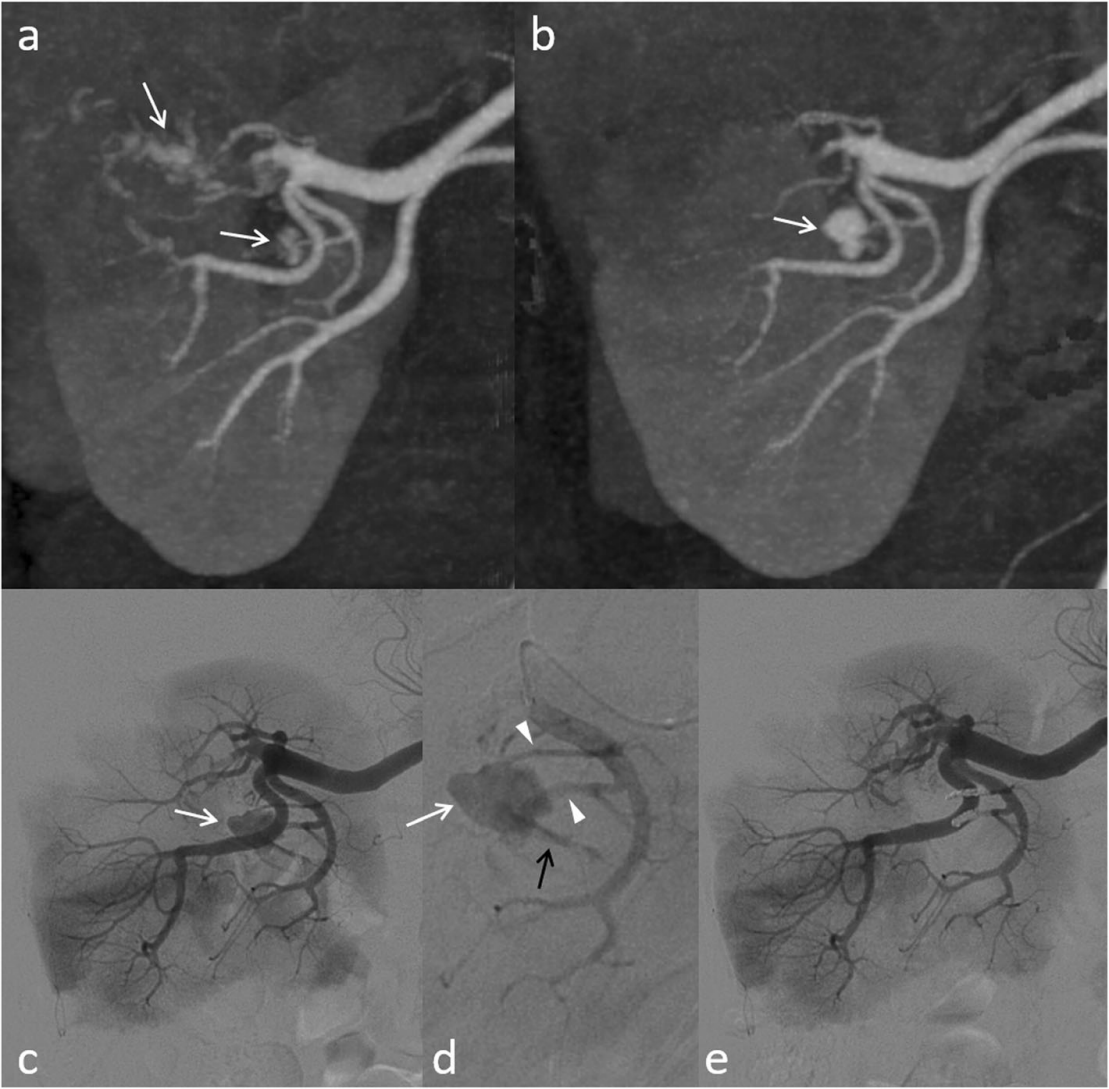
A case of a developing renal artery pseudoaneurysm (RAP), with symptoms, in a 54-year-old woman. **a** Irregularly shaped enhancements are observed (arrows) on early postoperative contrast-enhanced CT on postoperative day (POD) three. **b** The larger one disappeared and the smaller one increased in size developing into RAP (arrow) on CT on POD seven, with sudden hematuria. **c**, **d**, **e** Angiography shows two branches (arrowheads) that feed the RAP (white arrows) with extravasation into the renal pelvis (black arrow), which are successfully embolized using microcoils on POD eight

**Fig. 5:**
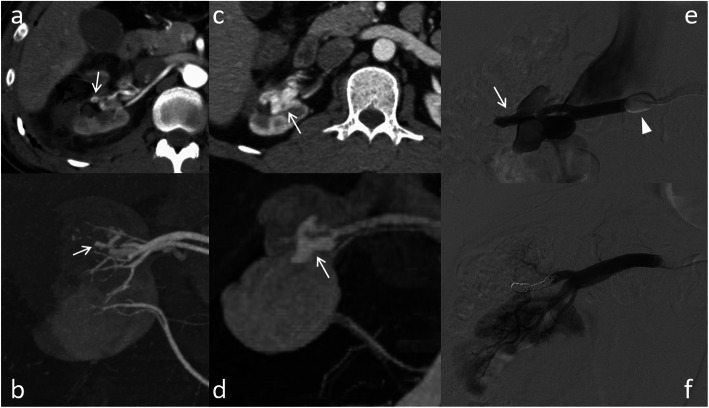
A case of an increasing arteriovenous fistula (AVF), without symptoms, in a 40-year-old man. **a**, **b** Irregularly shaped enhancement (arrows) is suspected on early postoperative contrast-enhanced CT on postoperative day (POD) four. **c**, **d** It is increased in size and AVF is suspected (arrows) on follow-up CT after 1 year without symptoms. **e** The AVF (arrow) is embolized using microcoils under balloon occlusion (arrowhead) on POD 414

Among the 8 patients with emergent TAE after scheduled early CE-CT, one patient underwent TAE on POD 21 with hematuria for AVFs that were not detected on early CE-CT on POD two (Fig. 3). Apart from this case, early CE-CT showed some findings, as follows: irregular enhancement 5, AVF 1, and a minute RAP of 3 mm 1. A typical case of a developing RAP with symptoms is shown in Fig. 4. Among the 64 patients with elective TAE, one patient underwent TAE for increased AVF, without symptoms, on POD 414 (Fig. 5). In this patient, AVF was suspected on early CE-CT since POD four. However, TAE was not performed on that day. Except for this case, TAE was performed during the postoperative period. The angiographic findings and embolization methods for RAP including these patients are summarized in Table 2.

**Table 2:** Angiographic findings and embolization methods

No. of lesions	1.7 ± 0.9 (total 139)
Shape of lesions	Oval-round 60, oval-round + AVF 36, irregular + AVF 14, AVF 12, irregular 10, disruption 4, and extravasation 3
Size of RAP (mm)	12.8 ± 6.0
No. of embolized branches	2.1 ± 1.2 (total 168)
Level of embolized branches	1st branch 0, 2nd branch 25, 3rd branch or more 143
Microcoils	4.6 ± 3.1 (total 376) (Target 116, Penumbra 35, GDC 17, Interlock 145, IDC 4, Tornado 59)
Other embolic materials	Gelatin sponge 27, NBCA 6
Amount of contrast material (mL)	74.7 ± 32.4

*AVF* Arteriovenous fistula, *NBCA* n-butyl-2-cyanoacrylate, *RAP* Renal artery pseudoaneurysm

The complications after TAE are summarized in Table 3. No major complications were observed. Minor complications were observed in 20 (24.4%) patients. All of them were mild and disappeared with conservative therapy. All fever was subsequent, after surgery fever. Regarding pain, half of it was back pain due to bed rest after TAE. The median inpatient days after TAE was two (range, 1–14). Thirty-nine patients (47.6%) were discharged on the day immediately after TAE. The laboratory data are summarized in Table 4. Although the most recent eGFR after surgery was significantly lower than the pre-surgery rate (*p* < 0.001), no deterioration of the eGFR was observed from pre- to post-TAE (*p* = 0.902) or from pre-TAE to the most recent measurement (*p* = 0.090). No patient required dialysis initiation after surgery or TAE.

**Table 3:** Complications after TAE

Major complications	0 (0%)
Minor complications	20 (24.4%)
Back pain (apart from surgical pain)	8 (9.8%)
Fever (more than 3 days)	8 (9.8%)
Vagal reflux	3 (3.7%)
Femoral hematoma	1 (1.2%)

*TAE* Transarterial embolization

**Table 4:** Changes in laboratory data

	Pre-surgery	Pre-TAE	Post-TAE on the next day	Most recent 1284 (5–2958) days
WBC (10^3^/μL)	5761 ± 1623	7635 ± 3016	7746 ± 2269	5308 ± 1370
CRP (mg/dL)	0.15 ± 0.31	6.53 ± 5.12	3.98 ± 3.75	0.16 ± 0.26
LDH (U/L)	181.5 ± 29.1	285.4 ± 94.6	277.4 ± 93.4	186.1 ± 34.8
eGFR (mL/min/1.73 m^2^)	72.7 ± 15.7^*^	64.6 ± 18.6^†,‡^	64.2 ± 18.4^†^	60.2 ± 13.7^*,‡^

*TAE* Transarterial embolization, *WBC* White blood cell, *CRP* C-reactive protein, *LDH* Lactate dehydrogenase, *eGFR* Estimated glomerular filtration rate

*P*-value = ^*^ < 0.001, ^†^0.902, and ^‡^0.090

## Discussion

We performed CE-CT in the early postoperative period, mainly 2 to 3 days after partial nephrectomy, to screen for RAP. This is done to prevent delayed hemorrhages, which usually occur approximately 2 weeks after surgery, by performing TAE before any ruptures. We show screening by early postoperative CE-CT can detect RAP which contributes to preventing delayed hemorrhage after partial nephrectomy (Morita et al. 2015). In half of the patients who underwent emergent TAE, symptoms presented on median POD two, before obtaining early postoperative CE-CT during hospitalization. In the other patients, except for one individual with a newly appearing AVF (Fig. 3), some findings, such as irregular enhancement, a minute RAP, and AVF, were observed on early postoperative CE-CT. Thus, delayed hemorrhage could be expected and TAE was performed smoothly. These factors lead to the successful outcome in which no patient died or had sequela due to bleeding. It is essential to perform less invasive surgeries in laparoscopic or robot-assisted laparoscopic partial nephrectomies.

It is debatable whether early postoperative CE-CTs are required. For the duration of the period analyzed in our study, the procedure was performed in every patient if possible. This is because we did not know the frequency of and risk factors for RAP and, in the dawn of laparoscopic or robotic partial nephrectomy in our institution, we desired to prevent hemorrhagic complications. However, CT radiation exposure and the adverse effects of contrast materials on the renal function in patients with partial nephrectomy are relevant concerns. Recently, we have only been performing early postoperative CE-CT in high-risk cases under the following circumstances: renal sinus exposure surgery, decrease in hemoglobin or blood pressure, or severe hematuria. In addition, we are now largely reducing the contrast material and radiation exposure dose by using a full iterative reconstruction method, a forward-projected model-based iterative reconstruction solution (FIRST; Canon Medical Systems, Otawara, Japan), while improving the visualization of the peripheral renal arteries (Morita et al. 2020). Furthermore, the frequency of RAP in our institution has recently decreased due to improvements in surgical techniques, such as early unclamping, avoidance of deep excision into the renal sinus, and the non-renorrhaphy technique (Omae et al. 2015; Tachibana et al. 2018; Kondo et al. 2015). However, the management of RAPs after partial nephrectomy should be carefully considered, especially in non-high-volume centers, because they can lead to life-threatening complications.

We have illustrated that TAE has no significant adverse effect on renal function. A recent report also showed no deteriorating effect on eGFR in 17 patients who underwent TAE when compared to 34 who did not, at a follow-up of 6 months (Baboudjian et al. 2020). In our study, the eGFR most recently recorded on the median POD 1284 was lower than the pre-surgery one. This is partly natural because the eGFR gradually decreases with age. In addition, the effect of partial nephrectomy is greater than that of TAE. This successful result is due to the development of devices for TAE. In the kidney, unlike the liver, infarction certainly occurs if normal arteries are embolized. The RAP feeders are mainly the third or more peripheral branches. They are thin and often multiple. Recent detachable microcoils, such as the Target Coil (Stryker Inc.) and the Penumbra Coil (Penumbra Inc), are ideal for embolizing these arteries because the coils are smaller, softer, and easier to position than the older pushable coils. In addition, we prefer to use a triaxial microcatheter system, such as the 1.9-F Carnelian Marvel (Tokai Medical Products), through a high-flow microcatheter. A higher flow rate is required for renal as opposed to liver angiography, especially for patients with AVF. High-flow microcatheters are ideal for obtaining a sufficiently accurate angiography and can support the inner microcatheter during coil embolization (Shimohira et al. 2013). In patients with renal insufficiency, preserving the normal kidney as much as possible and reducing the contrast material dose is crucial for maintaining renal function.

This study had several limitations due to its retrospective nature, with data collected from a single center. First, the study did not have a comparison group, with patients without early postoperative CE-CT and TAE, for example. We cannot know whether TAE is required in patients without symptoms. Over-treatment should be considered. However, it is difficult to not treat RAPs or AVFs if they are apparent. Although in rare cases bleeding due to RAP disappears spontaneously, Hyams et al. reported that the rate of resolution with conservative management alone was 11% (Hyams et al. 2011; Darbyshire et al. 2014). Further studies are required to investigate what kind of RAPs or AVFs can in the future be managed with conservative treatment.

## Conclusions

In conclusion, TAE is largely effective and safe for patients with bleeding or RAP, including AVF, screened by early postoperative CE-CT after partial nephrectomy. If embolization was performed adequately, no recurrence of bleeding or adverse effects on the renal function were observed in the mid-term follow-up period. However, AVF should be considered because it can develop infrequently, in a short or long period of time.

## Data Availability

The datasets used and/or analysed during the current study are available from the corresponding author on reasonable request.
